# Supplementary material for: Effects of a blend of essential oils in milk replacer on performance, rumen fermentation, blood parameters, and health scores of dairy heifers
Source: PLoS One. 2021 Mar 11;16(3):e0231068. doi: 10.1371/journal.pone.0231068 (PMC7951862; doi:10.1371/journal.pone.0231068)
Supplement: Attachment — Submitted filename: Manuscript ID PONE-D-20-07435.docx [file pone.0231068.s002.docx]

**Manuscript Number PONE-D-20-07435**

**Effects of a blend of essential oils in milk replacer on performance, rumen**

**fermentation, blood parameters and health scores of dairy heifers**

The authors present in the manuscript an exciting and original research idea relevant to the performance of dairy calves, and reserves to be published.

In general, the manuscript is well written, the statistical analyses are appropriate, and different parts are well presented and explained. After careful review of the document, the reviewer has the following minor suggestions:

**ABSTRACT**

Consider reporting the specific P-value instead of a general P ≤ 0.001 or P ≤ 0.05.

**MATERIALS AND METHODS**

Line 140: essential oils instead of essential oil.

Line 293: define performance. Does it mean BW and body measures?

**RESULTS AND DISCUSSION**

Similar to the abstract section, consider reporting the specific P-value in the text instead of a general P ≤ 0.001 or P ≤ 0.05.

Line 334: inverse association…, were. Did you mean where?

Line 341 – 351: consider adding of discussing why the lack of difference in responses in your study compared with those cited.

The reviewer considers that the authors should present the fatty acid composition or profile, at least the major fatty acids present in the BEO. It is a fundamental analysis to include in the manuscript and accounts for in the results and discussion section.
