# Supplementary material for: Effects of a blend of essential oils in milk replacer on performance, rumen fermentation, blood parameters, and health scores of dairy heifers
Source: PLoS One. 2021 Mar 11;16(3):e0231068. doi: 10.1371/journal.pone.0231068 (PMC7951862; doi:10.1371/journal.pone.0231068)
Supplement: Attachment — Submitted filename: ID PONE-D-20-07435_R2.docx [file pone.0231068.s003.docx]

**Manuscript Number PONE-D-20-07435**

**Effects of a blend of essential oils in milk replacer on performance, rumen**

**fermentation, blood parameters, and health scores of dairy heifers**

The authors significantly improved the manuscript. After reviewing the new version of the manuscript, the reviewer has the following minor suggestions:

**ABSTRACT**

Line 56: do not start sentences with abbreviations. The sentence could begin with The BEO…

**MATERIALS AND METHODS**

Line 56: do not start sentences with abbreviations, “The concentrations of NDF and ADF were determined…” instead. Similar on lines 194, 218, 232, 238, 493.

Line 157: 15 mL instead 15 ml.

Line 206: incomplete sentence…?

Line 227: remove the “d” after 90.

Line 289: Tween 80 should be capitalized since it is the name of a commercial product.

**RESULTS AND DISCUSSION**

Line 338 – 340: the sentence is difficult to follow. Consider paraphrasing it.

Line 341: it seems the word oil is missing after “cinnamaldehyde essential”

Line 423: the words “treatment effect” at the end of the sentence seem to be redundant.
