# Supplementary material for: Effects of a blend of essential oils in milk replacer on performance, rumen fermentation, blood parameters, and health scores of dairy heifers
Source: PLoS One. 2021 Mar 11;16(3):e0231068. doi: 10.1371/journal.pone.0231068 (PMC7951862; doi:10.1371/journal.pone.0231068)
Supplement: Attachment — Submitted filename: Response to reviewers.docx [file pone.0231068.s004.docx]

PONE-D-20-07435R1
Effects of a blend of essential oils in milk replacer on performance, rumen fermentation, blood parameters, and health scores of dairy heifers
PLOS ONE

Dear Dr. Palhares Campolina,

Thank you for submitting your manuscript to PLOS ONE. After careful consideration, we feel that it has merit but does not fully meet PLOS ONE’s publication criteria as it currently stands. Therefore, we invite you to submit a revised version of the manuscript that addresses the points raised during the review process.

THERE ARE SOME MINOR ISSUES THAT STILL NEED TO BE FIXED.

Please submit your revised manuscript by Nov 05 2020 11:59PM. If you will need more time than this to complete your revisions, please reply to this message or contact the journal office at plosone@plos.org. When you're ready to submit your revision, log on to https://www.editorialmanager.com/pone/ and select the 'Submissions Needing Revision' folder to locate your manuscript file.

Please include the following items when submitting your revised manuscript:

A rebuttal letter that responds to each point raised by the academic editor and reviewer(s). You should upload this letter as a separate file labeled 'Response to Reviewers'.

A marked-up copy of your manuscript that highlights changes made to the original version. You should upload this as a separate file labeled 'Revised Manuscript with Track Changes'.

An unmarked version of your revised paper without tracked changes. You should upload this as a separate file labeled 'Manuscript'.

If you would like to make changes to your financial disclosure, please include your updated statement in your cover letter. Guidelines for resubmitting your figure files are available below the reviewer comments at the end of this letter.

If applicable, we recommend that you deposit your laboratory protocols in protocols.io to enhance the reproducibility of your results. Protocols.io assigns your protocol its own identifier (DOI) so that it can be cited independently in the future. For instructions see: <http://journals.plos.org/plosone/s/submission-guidelines#loc-laboratory-protocols>

We look forward to receiving your revised manuscript.

Kind regards,

Juan J Loor
Academic Editor
PLOS ONE


Reviewers' comments:

Reviewer's Responses to Questions

**Comments to the Author**

1. If the authors have adequately addressed your comments raised in a previous round of review and you feel that this manuscript is now acceptable for publication, you may indicate that here to bypass the “Comments to the Author” section, enter your conflict of interest statement in the “Confidential to Editor” section, and submit your "Accept" recommendation.

Reviewer #1: (No Response)

Reviewer #2: All comments have been addressed

2. Is the manuscript technically sound, and do the data support the conclusions?

The manuscript must describe a technically sound piece of scientific research with data that supports the conclusions. Experiments must have been conducted rigorously, with appropriate controls, replication, and sample sizes. The conclusions must be drawn appropriately based on the data presented.

Reviewer #1: Yes

Reviewer #2: Yes

3. Has the statistical analysis been performed appropriately and rigorously?

Reviewer #1: Yes

Reviewer #2: Yes

4. Have the authors made all data underlying the findings in their manuscript fully available?

The [PLOS Data policy](http://www.plosone.org/static/policies.action#sharing) requires authors to make all data underlying the findings described in their manuscript fully available without restriction, with rare exception (please refer to the Data Availability Statement in the manuscript PDF file). The data should be provided as part of the manuscript or its supporting information, or deposited to a public repository. For example, in addition to summary statistics, the data points behind means, medians and variance measures should be available. If there are restrictions on publicly sharing data—e.g. participant privacy or use of data from a third party—those must be specified.

Reviewer #1: No

Reviewer #2: Yes

5. Is the manuscript presented in an intelligible fashion and written in standard English?

PLOS ONE does not copyedit accepted manuscripts, so the language in submitted articles must be clear, correct, and unambiguous. Any typographical or grammatical errors should be corrected at revision, so please note any specific errors here.

Reviewer #1: Yes

Reviewer #2: Yes

6. Review Comments to the Author

Please use the space provided to explain your answers to the questions above. You may also include additional comments for the author, including concerns about dual publication, research ethics, or publication ethics. (Please upload your review as an attachment if it exceeds 20,000 characters)

Reviewer #1: Overall, the majority of the edits were completed by the authors and have made improvements to the manuscript. There are still a few areas that need to be modified or clarified to be resolved and are detailed below. Some conclusions and supporting citations do not necessarily fit with some of your results.
Line 53: delete “in”

**Au: Edited. Thank you (L. 53)**

Line 56-57: replace “to” with “in” after both “ruminal manipulation” and “carryover effects”.

**Au: Edited. Thank you (L. 56-57).**

Line 61-63: Suggest “A good calf rearing program should embrace aspects that encompass body development, stress reduction, meet nutritional requirements, and housing management to optimize calf health status.”

**Au: Thank you for your suggestion. Edited (L.61-63).**

Line 64: suggest “essential” should be “key”

**Au: Edited. Thank you (L. 64).**

Line 70: “Raising” should be “raise”

**Au: Edited. Thank you (L. 70).**

Line 73: Please include reference after statement regarding rumen development beginning with “Additionally”.

**Au: Thank you for your concern. We included the reference in the statement (L.75).**

Line 75: Grammar edit- “too” should be “to”; also add “to” after “adapt”.

**Au: Edited. Thank you (L. 75).**

Line 80: “Wildly” should be “widely.”

**Au: Edited. Thank you (L. 80).**

Lines 80-88: Antibiotic growth promoters and antibiotics used for treatment of acute onset of illness are not the same. The statement that 90% of dairy farms provide AGPs for disease prevention (Line 86) is not correct, for several reasons. First, the publication from which you reference this percentage uses data collected from the mid to late 2000s, and was published prior to the USDA’s Veterinary Feed Directive in 2015. Under the VFD, use of AGPs is no longer permitted on US dairy farms without Veterinary supervision (nor in most other countries). Second, according to the literature referenced, this number refers to the percentage of dairy farms in the US that administer antibiotics at dry-off to prevent intramammary infection during the dry period- which is not the same as an AGP. I also caution you about your reference to 80% of all antibiotics used in the country are from livestock production (Line 84); this number was calculated by ‘public health advocacy groups’ and is cautioned against in the publication by the FDA. Many glaring inconsistencies exist in data attempting to find the source of antibiotic overuse, and the reality is that judicious use of antimicrobials needs to happen across all health sectors, not just one. Also not recommended to use the word “abuse” (Line 88); suggest ‘overuse’ instead.
Suggest revising and truncating this paragraph to focus on the motivation to find alternatives to antibiotic use due to growing concerns of resistance and ineffectiveness, and structure it more to lead into the exploration of phytogenic agents as alternatives.

**Au: Thank you for your concern. We did an intense literature review and we re-wrote the entire paragraph. Your comment is extremely pertinent, and we changed the previous statements. Thank you for your contribution (L.80-103).**

Line 92: extra space between “first line”

**Au: Thank you for your concern. With the paragraph review, this sentence has been changed.**

Line 95:- Deleted “The” at the beginning of the sentence

**Au: Edited. Thank you (L. 104).**

Line 100: “change” should be “by changing”

**Au: Edited. Thank you (L. 110).**

Line 101-102: reference for this statement?

**Au: Thank you for your concern. We added the reference for the statement (L. 111).**

Line 103-104: “Improving” should be “improve”; “decreasing” should be “decrease”.

**Au: Edited. Thank you (L. 113).**

Line 105: Change “focus” to “focusing”. Add apostrophe after “oils”. Consider doing additional search to find more data on essential oil supplementation in swine and poultry to further supplement this section if you want to continue to make comparison.

**Au: Edited. Thank you. We also added additional data of essential oils use for swine and poultry (L. 114-121).**

Line 108: Remove apostrophe from “molecules” and place on “oils”.

**Au: Edited. Thank you (L. 123).**

Line 113: Add “if” after “evaluate”.

**Au: Edited. Thank you (L. 127).**

Line 134: “hours” should be “h”s

**Au: Edited. Thank you (L. 148).**

Line 140: “minutes should be “min”

**Au: Edited. Thank you (L. 153).**

Line 141: Change “pipped” to “piped”.

**Au: Edited. Thank you (L. 154).**

Line 151: delete “New Zealand” already stated above so don’t need to state again.

**Au: Edited. Thank you (L. 164).**

Line 166 Table 1 and line 189: If you have weekly composite samples of each feedstuffs for analysis you should include or have a standard deviation for your nutrient analysis. Please include or restate how you analyzed your feedstuffs for this experiment. Add to Statistical Analysis section

**Au: Thank you for your concern. We calculated the weekly composite samples' standard deviations and added to the table (L 177).**

Line 181: should be “fixed for a maximum…”

**Au: Edited. Thank you (L. 193).**

Line 184: add “treatment” after anti-inflammatory.

**Au: Edited. Thank you (L. 196).**

Line 188: “was” should be “were”

**Au: Edited. Thank you (L. 200).**

Line 189: “week” should be “weekly”

**Au: Edited. Thank you (L. 201).**

Line 199: Start sentence with “Body weight”

**Au: Edited. Thank you (L. 212).**

Line 206: Sentence is incomplete.

**Au: Thank you for your concern. We added the missing part of the sentence (L. 219).**

Line 224 to 225: delete “after that,”

**Au: Edited. Thank you (L. 237).**

Line 252: suggest “calculated [27]. In addition, platelet… (NLR) were calculated”

**Au: Edited. Thank you (L. 266- 267).**

Line 262: Remove “severe” as it is redundant.

**Au: Edited. Thank you (L. 275-276).**

Line 277: Suggest adding “consecutive” after two and delete sequential

**Au: Edited. Thank you (L. 291).**

Line 283-294: It is unclear why a 1 microgram/mL concentration was chosen as your concentration to test the MIC. If my calculations are correct 1 g of BEO= 1,000,000 micrograms. When 1,000,000 micrograms are divided by the fluid volume of milk replacer provided per day to each calf (5,000 mL) this is 200 micrograms per ML for the dosage rate, yet you tested 1 microgram/mL. Could you explain in more detail why the concentration that was used for MIC was done and how this relates to the your actual feeding rate in the experiment. Suggest discussion in your results and discussion section.

**Au: Thank you for your concern. Your calculations are right, and the dose used in the MIC was wrongly expressed. We wanted to say that we started with an initial concentration of 1.0 mg/mL (Five times higher than the one used for the calves), and then we diluted it from 1:2 until 1:256. We changed to the correct measure unit. We are sorry for the misunderstanding (L. 305).**

Line 293: extra space after 35

**Au: Edited. Thank you (L. 307).**

Line 315: Italicize P in P-value

**Au: Edited. Thank you (L. 329).**

Line 330-331: Suggest “compromised” should be “limited and “pre-weaning” might be better suited to describe as first 30 d of life when intake of starter will be low so the desired supplementation level may not be achieved based on intake levels of the starter.

**Au: Thank you for your concern. The suggestion makes the statement clearer. Edited (L. 344-346).**

Line 331: suggest deleting “it was decided to offer” and add “was offered” after BEO

**Au: Edited. Thank you (L. 346).**

Line 333: “consequently” should be “subsequently”

**Au: Edited. Thank you (L. 348).**

Line 334: suggest deleting sentence starting with “there was no rejection” and replacing

“with no refusal and good acceptance” with “indicating no palatability issues of BEO”

**Au: Edited. Thank you (L. 349).**

Line 337: delete “way of”

**Au: Edited. Thank you (L. 351).**

Line 340: What do you mean by “young animals x old animals”?

**Au: Thank you for your concern. We changed the statement to make a clear statement. By young animals, we mean juvenile animals, and old animals, we mean adults (L. 307).**

Line 344: I think you need to end this paragraph by restating that in this study palatability was not a problem with the mixture used.

**Au: Thank you for your concern. We added a sentence at the end of the paragraph to emphasize the response as you suggested (L. 359-360).**

Line 348 and 394- Don’t need n= 29 because of number below in table.

**Au: Edited. Thank you (L. 364 and 409).**

Table 2- for MR intake in because of how you analyzed and presented the CI I don’t think SEM should be included in the table.

**Au: Edited. Thank you. We removed the SEM (L. 365).**

Line 358- Suggest “An observed effect between fecal scores…” Although I am not sure this correlation really adds to your point. It seems like your objective is to determine a treatment by time interaction of your variables in response to BEO which can be described for both MR intake and fecal scores already without a further correlation between the two. As a reader I think I would be ok with you stating that MR intake was lower during certain weeks (if significant) and also fecal scores were elevated during those same weeks which are likely related because intake decreases when animals are sick.

**Au: Thank you for your concern. Besides, it is well known that intakes decrease with sickness, we did the correlation just to statistically prove that the association between intake and the fecal score was negative (L. 373 and 375).**

Line 367- according to table 3 feed efficiency in the preweaning period tended to be lower in calves supplemented with BEO but no difference postweaning. On Line 375 you make about postweaning feed efficiency carry over effects but there does not appear to be any carryover effects in terms of intake or feed efficiency in your experiment. It is unclear what connections and conclusions you are trying to make here.

**Au: Thank you for your concern. The statement on line 375 referend to carry-over effects in other studies and not on this one. However, we reformulate the sentence to be clear. (L. 391-394).**

Line 380: Correct spelling of “monensin”

**Au: Edited. Thank you (L. 397).**

Line 383: Replace “masquerade” with “mask”

**Au: Edited. Thank you (L. 400).**

Line 384-385: Suggest “In this study, no antagonism between additives was observed, as there were no negative responses for BEO compared to CON”

**Au: Edited. Thank you (L. 401-403).**

Table 3: “Kg/Kg” should be “kg/kg” for both feed efficiencies

**Au: Edited. Thank you.**

Line 403-406: Your comment about structural growth being related to protein in the diet in starter- how does this relate to protein provided (MR and starter) and consumed by your calves related to their expected requirements or growth. By bringing in this information are you saying your calves had already met their requirements for optimal growth or that they were limited on protein so you might not expect a response?

**Au: Thank you for your concern. We formulated the diet and increased the solids in the milk replacer to meet the requirement to our calves for optimal growth. However, we did not test different protein levels in the starter or in the milk replacer to see their interaction with the essential oil supplementation. We added a statement to the manuscript to clarify this point (L.424-426).**

Line 410: “developed” instead of “develop”

**Au: Edited. Thank you (L. 429).**

Line 417: Wrong P-value listed in text; this is the pre-weaning week effect P-value. Your actual P-value for this variable was only a tendency so make sure you adjust your wording and conclusions related to this appropriately.

**Au: Thank you for your concern. We corrected the p-value on the text. We considered statistically different when P ≤ 0.05, and we did not work with tendencies (L. 437).**

422-424- it is unclear what this sentence contributes

**Au: Thank you for your concern. The sentence was relocated to a different place to contribute with the result discussion (L. 429).**

Table 4- The C2:C3 ratios presented in the table seem high based on the Acetic and Propionic values presented for example preweaning C2 was 30.8 and C3 was 18.88 which is a ratio of 1.63 not 1.97. Could you verify the numbers you presented are correct?

**Au: Thank you for your concern. We re-run the statistics and the values are correct. The results not presented in the manuscript are listed below.**

**C2:C3**

**Treatment	emmean	SE	df	lower.CL upper.CL**

**BEO	1.69	0.147	23	1.38	1.99**

**CON	1.97	0.133	23	1.69	2.24**

**Week**

**3	2.59	0.227	23	2.13	3.06**

**5	1.88	0.169	23	1.53	2.23**

**7	1.48	0.146	23	1.18	1.78**

**9	1.35	0.151	23	1.04	1.67**

**SEM = 0.119**

**CV = 17**

**Acetic**

**Treatment	emmean	SE	df	lower.CL upper.CL**

**BEO	27.16	2.012	23	22.99	31.32**

**CON	30.79	2.050	23	26.55	35.04**

**Week**

**3	17.15	2.838	23	11.17	22.92**

**5	30.35	2.358	23	25.47	35.23**

**7	33.27	2.022	23	29.09	37.45**

**9	35.25	2.100	23	30.91	39.60**

**SEM = 8.15**

**CV = 25.9**

**Propionic**

**Treatment	emmean	SE	df	lower.CL upper.CL**

**BEO	20.01	1.455	23	17.00	21.65**

**CON	18.18	1.339	23	16.11	23.02**

**Week**

**3	8.50	2.460	23	3.41	13.59**

**5	19.20	2.275	23	14.49	23.90**

**7	23.76	1.178	23	21.32	26.20**

**9	26.33	1.291	23	23.66	29.00**

**SEM = 7.11**

**CV = 33.2**

Line 436-439- I am not sure what these points really contribute to understanding what happened in response to the treatments you used and the results you collected

**Au: Thank you for your concern. We out the statement just to justify why our animals had low pH when compared to adult animals and that this low ruminal pH did not create any health problem.**

Line 44--: how are essential oils enhanced by low pH?

**Au. Thank you for your question. Some studies reported the there is a higher level of bioactivity at acidic pH. So, at low pH the essential oils behave in a more hydrophobic way and enter more easily inside the cells. This explains how the antimicrobial activity of the essential oils is enhanced at low pH.**

Line 446- your results don’t support decreased ruminal nitrogen ammonia or increases in propionate and butyrate

**Au: Thank you for your concern. Indeed, there were no statistical differences in ruminal ammonia or the individual VFA values. However, there was a statistical difference within the proportion between the VFA. Therefore, we believe that besides no statistical difference in the VFA alone, it could be a biological difference that could lead to the difference in the proportions.**

Line 456 to 457- it is not clear the values that you are presenting in this statement or how they relate to what you did. Are these values form a different experiment? What does the P-value relate to? If it is a different study you shouldn’t report the actual P-value. Need more context.

**Au. Thank you for your concern. This statement refers to another experiment. We have reformulated the statement to the manuscript to clarify this point (L.473-475).**

Line 461- “This way of providing it” suggest should be “By providing the BEO in the MR, the treatment should be diverted past the rumen and have minimal impact on local ruminal microbiota and VFA.”

**Au: Thank you for your suggestion. Added (L.479-481)**

Line 465- delete “to”

**Au: Edited. Thank you (L. 484).**

Line 468: “it is shown a direct effect” should be “supplementation of essential oils have shown a direct effect”

**Au: Thank you for your suggestion. Added (L.487).**

Line 483- Suggest” For all ruminal parameters, a week effect during preweaning was observed”

**Au: Thank you for your suggestion. Added (L.503).**

Line 515- Suggest “All blood cell counts were within normal ranges based on age and species

**Au: Thank you for your suggestion. Added (L.535)**

Line 521- delete “are” and “originated” should be originate

**Au: Edited. Thank you (L.541).**

Line 526 to 527 and line 529 to 530: need context about which treatment you are talking about

**Au: Thank you for your suggestion. We added more information to make a clearer statement (L.547-548).**

Line 546: delete “An”

**Au: Edited. Thank you (L.566).**

Line 562: Reference needed

**Au: Added. Thank you (L.584).**

Line 567: BEO shouldn’t be used here because that is your treatment description whereas this is a generalization of other blends of essential oils. Reference for this statement?

**Au: Thank you for your concern. BEO is our treatment description. We changed the statement to be clearer. We added the reference. (L.587, 589)**

Line 572: add P-value for days with diarrhea. And Percent of calves with diarrhea (Line 572-574)

**Au: Added. Thank you (L.592 and 594).**

Line 575: should be changed over time

**Au: Edited. Thank you (L.595).**

Line 576: “within” should be “between” and add P-value

**Au: Edited. Thank you (L.596-597).**

Line 581: Replace “it is hard to point out” with “it is important to point out” and remove “since”.

**Au: Thank you for your suggestion. Edited. (L.602-603).**

Table 7: Pre-weaning period is listed as 4-60 d previously; change 61 to 60 for consistency.

**Au: Edited. Thank you (Table 7).**

Line 596: what about avg days for CON?

**Au: Thank you for your concern. The average was the same for both treatments. However, we added to make a clearer statement (L.617).**

Line 616: Capitalize Gram.

**Au: Edited. Thank you (L. 637).**

Reviewer #2: The reviewer thanks the authors for addressing the comments raised in the previous review. However, there are few more comments or suggestions to improve the presentation of the manuscript.

**Manuscript Number PONE-D-20-07435**

**Effects of a blend of essential oils in milk replacer on performance, rumen**

**fermentation, blood parameters, and health scores of dairy heifers**

The authors significantly improved the manuscript. After reviewing the new version of the manuscript, the reviewer has the following minor suggestions:

**ABSTRACT**

Line 56: do not start sentences with abbreviations. The sentence could begin with The BEO…

**Au: Edited. Thank you (L. 156).**

**MATERIALS AND METHODS**

Line 56: do not start sentences with abbreviations, “The concentrations of NDF and ADF were determined…” instead. Similar on lines 194, 218, 232, 238, 493.

**Au: Edited. Thank you (L. 206, 230, 244-245, 251, 506-507).**

Line 157: 15 mL instead 15 ml.

**Au: Edited. Thank you (L. 170).**

Line 206: incomplete sentence…?

**Au: Thank you for your concern. It was missing part of the sentence and we added to complete the statement (L. 218).**

Line 227: remove the “d” after 90.

**Au: Edited. Thank you (L. 239).**

Line 289: Tween 80 should be capitalized since it is the name of a commercial product.

**Au: Edited. Thank you (L. 303).**

**RESULTS AND DISCUSSION**

Line 338 – 340: the sentence is difficult to follow. Consider paraphrasing it.

**Au: Thank you for your concern. We changed the sentence to make a clear statement (L. 352-354).**

Line 341: it seems the word oil is missing after “cinnamaldehyde essential”

**Au: Added. Thank you (L. 303).**

Line 423: the words “treatment effect” at the end of the sentence seem to be redundant.

**Au: Thank you for your concern. We have relocated this sentence and took the redundant words (L. 435-436).**
